# Entropic Uncertainty for Two Coupled Dipole Spins Using Quantum Memory under the Dzyaloshinskii–Moriya Interaction

**DOI:** 10.3390/e23121595

**Published:** 2021-11-28

**Authors:** Ahmad N. Khedr, Abdel-Baset A. Mohamed, Abdel-Haleem Abdel-Aty, Mahmoud Tammam, Mahmoud Abdel-Aty, Hichem Eleuch

**Affiliations:** 1 Department of Physics, Faculty of Science, Al-Azhar University, Assiut 71524, Egypt; A.N.khedr@azhar.edu.eg (A.N.K.); amabdelaty@ub.edu.sa (A.-H.A.-A.); tammam@azhar.edu.eg (M.T.); 2 Department of Mathematics, College of Science and Humanities in Al-Aflaj, Prince Sattam bin Abdulaziz University, Al-Aflaj 11942, Saudi Arabia; 3 Department of Mathematics, Faculty of Science, Assiut University, Assiut 71515, Egypt; 4 Department of Physics, College of Sciences, University of Bisha, Bisha 61922, Saudi Arabia; 5 Department of Mathematics, Faculty of Science, Sohag University, Sohag 82524, Egypt; mabdelaty@zewailcity.edu.eg; 6 Department of Applied Physics and Astronomy, University of Sharjah, Sharjah 27272, United Arab Emirates; heleuch@sharjah.ac.ae; 7 Department of Applied Sciences and Mathematics, College of Arts and Sciences, Abu Dhabi University, Abu Dhabi 59911, United Arab Emirates; 8 Institute for Quantum Science and Engineering, Texas A&M University, College Station, TX 77843, USA

**Keywords:** quantum-memory-assisted entropic uncertainty, entanglement, mixedness, Dzyaloshinskii–Moriya interaction, dipolar system

## Abstract

In the thermodynamic equilibrium of dipolar-coupled spin systems under the influence of a Dzyaloshinskii–Moriya (D–M) interaction along the *z*-axis, the current study explores the quantum-memory-assisted entropic uncertainty relation (QMA-EUR), entropy mixedness and the concurrence two-spin entanglement. Quantum entanglement is reduced at increased temperature values, but inflation uncertainty and mixedness are enhanced. The considered quantum effects are stabilized to their stationary values at high temperatures. The two-spin entanglement is entirely repressed if the D–M interaction is disregarded, and the entropic uncertainty and entropy mixedness reach their maximum values for equal coupling rates. Rather than the concurrence, the entropy mixedness can be a proper indicator of the nature of the entropic uncertainty. The effect of model parameters (D–M coupling and dipole–dipole spin) on the quantum dynamic effects in thermal environment temperature is explored. The results reveal that the model parameters cause significant variations in the predicted QMA-EUR.

## 1. Introduction

The Heisenberg uncertainty relation [1] has been extensively explored experimentally and theoretically because of its high ability to distinguish between the boundaries of classical and quantum mechanics. According to Heisenberg’s uncertainty relation, it is impossible to know a particle’s position and momentum with high precision at the same moment [2]. Therefore, an entropic-uncertainty relation was proposed [3,4,5]. However, the association between uncertainty relations and other essential qualitative features, entanglement and coherence was first discussed in the Einstein–Podolsky–Rosen (EPR) article [6], but there were no quantitative useful criteria at that time. Uncertainty violations were implemented as a signature of entanglement [7,8,9]. Recently, new entropic inequalities for the different quantum systems using the phase-space probability representation of quantum states have been reported [10].

Using quantum memory, the uncertainty relations can be improved by substituting standard deviations with a closer entropic uncertainty relationship, leading to entropic uncertainty relations [11,12], which have been experimentally confirmed [13,14]. The relationships between entropic uncertainty in the presence of quantum memory were analyzed using the effect of the entanglement [15] between the state of the observed system and that of another quantum state (memory system) [16]. In the absence of entanglement, this uncertainty relation leads to Deutsch’s outcome [3]. The relations of uncertainty can be perceived as a game between Alice and Bob [17].

Over the last few years, several significant applications of QMA-EUR in the field of quantum processing information, such as witness of entanglement [18,19], cryptography [20,21], quantum-key distributions [22,23], quantum-speed limit [24,25], as well as quantum metrology [26], have been discovered. The impacts of different noise forms in nitrogen-vacancy centres in diamond on the dynamics of QMA-EUR were investigated experimentally [19,27,28,29]. The strong association between uncertainty relations and mixedness has been intensively analyzed [30,31]. The entanglement and the mixedness have been studied to identify how they affect the deterministic uncertainty tightness and the entropic uncertainty’s lowest bound [32,33]. The properties of the QMA-EUR in a spin chain model were studied in a uniform and irregular magnetic field with (D–M) interaction [34]. It was also explored in the Ising model under long range influence with an arbitrary-magnetic field [35]. The effect of decoherence on the dynamics of QMA-EUR and the quantum coherence of the system in the subject was reported [36,37].

Currently, quantum information theory is a branch of science that aims at characterizing and quantifying quantum correlations, particularly entanglement, to use in the modification, storage, and transmission of data [38]. By employing the quantitative nature of many physical systems, quantum information theory seeks a higher level of prosperity in its technological possibilities. Such systems are expected to be useful filters for usage in many areas of physics such as solid-state spins with optically interfaced quantum technologies [39] and quantum annealing processor [40,41]. The spin dipolar system is one of the promising prototypes of understanding the several phenomena in quantum systems, due to its ability to produce a sufficient number of qubits, their coherence for a long time, and the fine-tuning of their magnetic properties electronically. These aforementioned properties can be realized in many solid-state spin systems as quantum spin systems [42,43], rotational- states of molecules [44] and nitrogen vacancy-centers in diamond [45,46]. It has been demonstrated that, for dipolar spin interaction and 2-photon resonance between two qubits and a coherent cavity field, the dipolar interaction could contribute to resilience toward intrinsic decoherence and maintain a stronger correlations [47,48,49,50,51]. The dipole acts as a noise source in many physical systems, which degrades the quantum system properties [52,53,54]. Recently, the quantum dynamics of the two qubits have been studied qualitatively and quantitatively in the presence of Dzyaloshinskii–Moriya and dipole–dipole interactions [55], as well as external time-dependent magnetic fields [56].

To our knowledge, the exploration of the association between the quantum correlation phenomenon of thermal equilibrium with the deterministic uncertainty correlation in dipole–dipole spin systems and in the presence of the Dzyaloshinskii–Moriya interaction is limited. Therefore, it is essential to comprehend QMA-EUR behaviour in spin-dipole systems in a state of thermal equilibrium. In this paper, we will investigate the dynamical characteristics of QMA-EUR and its lower bound, quantum correlation, mixedness and tightness for a dipolar spin interaction between two qubits with the implications of D–M interaction on Pauli’s two measurements, when Bob and Alice participate in the quantum system for the quantum uncertainty game.

The paper is arranged, as follows: In Section 2, the model for dipolar coupled-spin systems containing D–M interaction is introduced, as well as the method for its solution. Some essential descriptions are used to review the issue of the QMA-EUR, the entropic uncertainty relation’s tightness, entanglement and mixedness in Section 3. Section 4 presents outcome discussions on the studied quantum measures. Section 5 is dedicated to the conclusions.

## 2. Dipole–Dipole Two-Spin System

Here, the considered system Hamiltonian describes dipole–dipole and Dzyaloshinsky–Moriya (D–M) interactions between two spins (identified by *A* and *B*). The dipole–dipole interaction is due to the action of the magnetic field generated throughout the magnetic moment of rotation on another spin at the nearest location [57,58]. Spin-orbit coupling causes the Dzyaloshinsky–Moriya interaction. The Hamiltonian of the dipole two-spin model is denoted by:

(1) $$\hat{H}=-\frac{1}{3}{\vec{\sigma }}_{1}\cdot {\vec{I}}_{\sigma_{2}}\cdot {\vec{\sigma }}_{2}^{t}+\sum_{j=x,y,z}D_{j}\left(\sigma_{A}^{j+1}\sigma_{B}^{j+2}-\sigma_{A}^{j+2}\sigma_{B}^{j+1}\right),$$

where ${\vec{\sigma }}_{k}=\left\{\sigma_{k}^{x},\sigma_{k}^{y},\sigma_{k}^{z}\right\}\left(k=A,B\right)$ represents the usual k-spin Pauli operator vector, ${\vec{I}}_{\sigma_{2}}=\left\{\left(\Delta -3\epsilon \right)\sigma_{B}^{x},\left(\Delta +3\epsilon \right)\sigma_{B}^{y},\left(-2\Delta \right)\sigma_{B}^{z}\right\}$. Δ and ϵ design the coupling constants of the 2-coupled dipole spins. The orientation of the spin depends on the sign of Δ. If the value Δ is less than zero, the rotation is in the *x*–*y* dimension. Otherwise, the orientation of spin is along the *z*-axis. $D_{j}\left(j=x,y,z\right)$ represents the components of the D–M coupling vector. Here, the study is limited to the state where the D–M coupling is represented individually along the *z*-axis, i.e., $D_{x}=D_{y}=0$. On the basis of B={|00〉,|01〉,|10〉,|11〉}, we can express the Hamiltonian (1) as:

(2) $$\begin{array}{ccc} \hat{H} & = & \frac{2}{3}\left[(\Delta (|00\rangle \langle 00\right|-\left|01\rangle \langle 01\right|-\left|10\rangle \langle 10\right|+|11\rangle \langle 11|)+6\epsilon (|00\rangle \langle 11|+|11\rangle \langle 00|)\\ & - & \left(\Delta +3iD_{z}\right)\left|10\rangle \langle 01\right|-\left(\Delta -3iD_{z}\right)|01\rangle \langle 10|] \end{array}$$

Using the condition of the eigenvalue-problem: $\hat{H}|S_{i}\rangle =V_{i}|S_{i}\rangle \left(i=1,2,3,4\right)$, the eigen-energy levels (the eigenvalues $V_{i}$ of the two-spin Hamiltonian) can be written as

(3) $$\begin{array}{ccc} V_{1} & = & \frac{2}{3}\left(\Delta -3\epsilon \right),\;V_{2}=\frac{2}{3}\left(\Delta +3\epsilon \right), \end{array}$$

(4) $$\begin{array}{ccc} V_{3} & = & \frac{2}{3}\left(-\Delta +\sqrt{\Delta^{2}+9D_{z}^{2}}\right),\;V_{4}=\frac{2}{3}\left(-\Delta -\sqrt{\Delta^{2}+9D_{z}^{2}}\right) \end{array}$$

The corresponding eigenvectors are:

(5) $$\begin{array}{c} |S_{1}\rangle =\frac{|1,1\rangle -|0,0\rangle }{\sqrt{2}}\;\;\;\;\;\;|S_{2}\rangle =\frac{|1,1\rangle +|0,0\rangle }{\sqrt{2}}\\ |S_{3}\rangle =\frac{|1,0\rangle -|0,1\rangle }{\sqrt{2}}\;\;\;\;\;\;\;|S_{4}\rangle =\frac{|1,0\rangle +|0,1\rangle }{\sqrt{2}} \end{array}$$

Therefore, it is simple to obtain the final density operator of a dipolar system at a thermal equilibrium:

(6) $$\begin{array}{ccc} \;\;\;\;\rho^{AB}\left(T\right) & = & \frac{1}{Z}e^{-H/K_{\beta }T}=\frac{1}{Z}e^{-V_{i}/K_{\beta }T}|S_{i}\rangle \langle S_{i}|\\ & = & \frac{1}{Z}\{e^{-\frac{1}{3}\left(2\beta \Delta \right)}\cosh\left(2\beta \epsilon \right)[|00\rangle \langle 00|+|11\rangle \langle 11|]-e^{-\frac{1}{3}\left(2\beta \Delta \right)}\sinh\left(2\beta \epsilon \right)\\ & & \times [|00\rangle \langle 11|+|11\rangle \langle 00|]+e^{\frac{2\beta \Delta }{3}}\cosh\left(\frac{2}{3}\alpha \beta \right)[|01\rangle \langle 01|+|10\rangle \langle 10|]\\ & & +\frac{1}{\alpha }e^{\frac{2\beta \Delta }{3}}\left(\Delta -3iD_{z}\right)\sinh\left(\frac{2}{3}\alpha \beta \right)|00\rangle \langle 11|+h.c.\}, \end{array}$$

where $\alpha =\sqrt{\Delta^{2}+9D_{z}^{2}}$, the partition function of the system $Z=2e^{-\frac{1}{3}\left(2\beta \Delta \right)}\left(e^{\frac{4\beta \Delta }{3}}\cosh\left(\frac{2\alpha \beta }{3}\right)+\cosh\left(2\beta \epsilon \right)\right)$, and $\beta =\frac{1}{KT}.$

## 3. Quantum Preliminaries of Relations

The deterministic uncertainty relation can be obtained by using quantitative memory of two incompatible measurements [11,16]

(7) $$S\left(X|B\right)+S\left(R|B\right)\geq S\left(A|B\right)+\log_{2}\frac{1}{c}$$

where $S\left(A|B\right)=S\left(\rho_{AB}\right)-S\left(\rho_{B}\right)$ is the conditional von Neumann entropy with $S\left(\rho^{AB}\right)=-Tr\left(\rho^{AB}\log_{2}\rho^{AB}\right)=-\sum_{j}\lambda_{j}\log_{2}\lambda_{j}$, $\lambda_{j}$ represent the system’s eigenvalues of $\rho^{AB}$. The parameter c on the right side of the inequality in Equation (7) is defined by $c=\max_{ij}{\left|\langle \phi_{i}|\varphi_{j}\rangle \right|}^{2}$ with $|\phi_{i}\rangle$ and $|\varphi_{i}\rangle$ are the observable’s eigenstates *X* and *R*. On the left side of the inequality in Equation (7), we define $S\left(Q|B\right)=S\left(\rho_{QB}\right)-S\left(\rho_{B}\right)$ with Q∈(X,R). Next, the quantum operation *A* is estimated by Q, the post-measurement case converts $\rho_{QB}=\sum_{i}\left(|\phi_{i}\rangle \langle \phi_{i}|⊗I\right)\rho_{AB}\left(|\phi_{i}\rangle \langle \phi_{i}|⊗I\right)$, where *I* is the identity operator. We use two Pauli observables, $\sigma^{x}$ and $\sigma^{z}$, as the measurement in this technique, with the eigenstates $|\sigma_{\pm }^{x}\rangle ={\left(1\;\;\;\pm 1\right)}^{T}/\sqrt{2}$, $|\sigma_{+}^{z}\rangle ={\left(1\;\;\;0\right)}^{T},|\sigma_{-}^{z}\rangle ={\left(0\;\;\;1\right)}^{T}$, respectively. Consequently, it is straightforward to get c=1/2. When *Q* and *B* are maximally entangled, it is possible to determine the entanglement memory results with certainty, i.e., there is no uncertainty.

### 3.1. Entropic Uncertainty

We adopted the following expression to examine the features of the entropy uncertainty rapport in accordance with the dipole interaction model, as follows.

The upper bounds of uncertainty relations (left-hand side (LHS)):

(8) $$\begin{array}{c} U_{L}=S\left(X|B\right)+S\left(R|B\right), \end{array}$$

are used to determine the accuracy of entropy uncertainty, while the uncertainty relations’ lower bound (right-hand side (RHS)) are utilized to determine the accuracy of the uncertainty in entropy from the relation:

(9) $$\begin{array}{c} U_{R}=\log_{2}\frac{1}{c}+H\left(A|B\right). \end{array}$$

### 3.2. Tightness

For convenience, the right and left sides of the uncertainty relation in Equation (7) are denoted by $U_{R}$ and $U_{L}$, respectively. Based on the proportion and disparity between the left and right sides of the uncertainty, $U_{d}$ and $V_{d}$ could be used to determine the degree of tightness of uncertain relationships:

(10) $$\begin{array}{c} U_{d}=\frac{S(X|B)+S(R|B)}{\left(\log_{2}\frac{1}{c}+S\left(A|B\right)\right)}\geq 1,\\ V_{d}=S\left(X|B\right)+S\left(R|B\right)-\log_{2}\frac{1}{c}-S\left(A|B\right). \end{array}$$

### 3.3. Quantum Information Resources

- Entanglement
  Here, the entanglement between the two dipole coupled spins is investigated using the concurrence [59], which is presented by:
  (11) $$C\left(\rho \right)=2\max\left\{0,C_{1}\left(\rho \right),C_{2}\left(\rho \right)\right\},$$
  where
  $$\begin{array}{c} C_{1}\left(\rho \right)=\sqrt{\rho_{14}\rho_{41}}-\sqrt{\rho_{22}\rho_{33}}\;,\;\;\;\;\;\;\;\;\;C_{2}\left(\rho \right)=\sqrt{\rho_{23}\rho_{32}}-\sqrt{\rho_{11}\rho_{44}}. \end{array}$$
  C=1, for the extreme entangled states and C=0, for the separable cases.
- Two-spin quantum coherence
  Based on the two-spin density matrix $\rho^{AB}\left(T\right)$ of Equation (6), the two-spin quantum coherence (mixedness) is investigated using the linear entropy [60], which can be given as:
  (12) $$L=\frac{d}{d-1}\left(1-Tr\left\{\rho^{AB}{\left(T\right)}^{2}\right\}\right).$$
  where *d* is the dimension of state $\rho^{AB}$. If L=0, the two-spin state is pure state. Otherwise, it has partial or maximal mixedness.

## 4. Results and Discussion

This section analyzes the relationships and the characteristics of the QMA-EUR, the tightness of uncertainty, the entanglement, and the mixedness for the dipolar spin system in thermodynamical equilibrium under the effects of the dipole–dipole and the Dzyaloshinsky–Moriya interactions.

**Case $D_{z}=0$**

**Figure 1:**
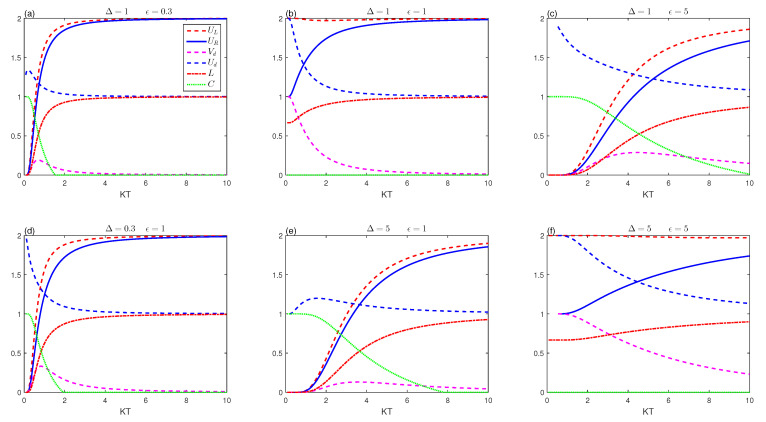
Entropic uncertainty $U_{L}$ (red-dashed line), the lower bound $U_{R}$ (blue-solid line), tightness uncertainty ($V_{d}$ (magenta-dashed line), $U_{d}$ (blue-dashed line)), the mixedness *L* (red dashed-dotted line) and concurrence (green-dotted line) against KT with $D_{z}=0$ for different Δ and ϵ.

Figure 1 reveals the dependence of the entropy uncertainty relation, the entanglement, and the quantum coherence on the normalized temperature KT, the dipolar coupling constants (Δ,ϵ) in the absence of D−−M interaction.

Figure 1a,b represent the uncertainty entropy, the linear entropy, the tightness of uncertainty and the concurrence measure against the normalized parameter KT for small values of the dipole coupling constant. It is clear that the behaviours of the entropic uncertainty $U_{L}$ (red dashed line), the lower bound $U_{R}$ (blue solid line), and the linear entropy *L* (red dashed-dotted line) vanish at low temperature (T→0) as shown in Figure 1a for a small amount of the coupling constant ϵ=0.3. Meanwhile, the two spins are in a maximally entangled state (see the green-dotted line). It is noticeable that the uncertainty rises to a constant value with increasing temperature, and the left side is always higher than the right side. In addition, as the temperature rises, the entanglement decreases to zero. After that, the phenomenon of the sudden death entanglement appears [61,62]. Moreover, since the mixedness reaches a fixed limit as the temperature rises, the increase of the mixedness enhances the entropic uncertainty, particularly for small values of the dipole interaction between the two spins. The mixedness is closely related to the uncertainty relation ($U_{L}$ and $U_{d}$), which presents an opposite behavior to the quantum entanglement. As a result, mixedness, like entanglement, may be utilized to comprehend the properties of uncertainty relations ($U_{L},U_{R}$). Aside from that, the deterministic uncertainty in the situation of convergent thermal equilibrium will be symmetrical at both low and high temperatures. Otherwise, $U_{L}$ will be greater than $U_{R}$. Based on $U_{L}$ and $U_{R}$, we can investigate the effects of the tightness of uncertain relationships for the difference between the inequality’s left and right sides $V_{d}$ (magenta-dashed line) as well as the ratio of the inequality’s left and right sides $U_{d}$ (blue-dashed line). The uncertainty relation $U_{L}$ = $U_{R}$ can be found for the equilibrium state. This indicates that the uncertainty relationships’ tightness has ended. However, when $U_{L}>U_{R}$, we observe a slight increase in the tightness $V_{d}$ of the uncertainty as it decreases to zero with the increase of the temperature. The function tightness $U_{d}$ of the uncertainty relation can be clearly defined by the ratio of the uncertainty of the two quantities and the entropic uncertainty relation’s lower bound as in Equation (10). We can see that the values of $U_{d}$ decrease as the temperature rises. This means that the increase of the temperature tightens the bounds of uncertain relationships.

Figure 1b depicts the deterministic uncertainty $U_{L}$ and its lower bound $U_{R}$, the mixedness entropy *L*, the function uncertainty tightness $\left(V_{d},U_{d}\right)$, and the concurrence for equivalent values of the dipole coupling constants (Δ=ϵ=1). It is clear that the functions $U_{L}$, $U_{R}$ and *L* have different initial values at relatively small temperatures (T≈0) due to the increase of the ϵ. The figure shows that the increase of the temperature leads to the increase of the amplifications of the uncertainties, the lower bound as well as mixedness. In this case, Δ=ϵ=1, the two-spin entanglement collapses for the equal values of the dipole two-spin interaction at $D_{z}=0$. The entropic uncertainty’s lower bound has a similar variability tendency as the entropic uncertainty. The lower bound and the mixed entropy have the same behaviours with different amplitudes and increase to their maximal value of two and one, respectively, as the temperature gets hotter. Clearly, there is a significant connection between mixedness and uncertainty in terms of behavior. The function tightness $U_{d}$ decreases inversely with the increase of the mixedness until both reach a constant level of stability. However, the tightness $V_{d}$ steadily decreases to zero as the temperature rises. This indicates that, as the temperature goes up, the uncertain relationships and the tightness of uncertainty become stronger. Meanwhile, we can observe that the concurrence evolution has a structure that is diametrically opposed to that of the mixedness entropy. Furthermore, the lower bound uncertainty has a well-established relationship with the tightness for an increasing temperature. When the lower bound is strong at higher temperature, the tightness is more tight. The given lower bound of the entropy uncertainty relationship is supported by the memory. With the aid of memory, the quantitative concurrence will influence the deterministic uncertainty relationship. The disappearance of the entanglement can cause the entropic uncertainty relationship to tighten. In this case Δ=ϵ=1, the increase of the mixedness improves the lower bound and the uncertainty tightness, which causes disappearance of the entanglement.

Figure 1c shows the effect of higher dipole coupling model parameters ϵ=5 for Δ=1 and $D_{z}=0$. For the lower temperatures, we observe that the higher values of the dipole coupling constants revive the quantum two-spin correlation. The green-dotted line curve shows that the increase of the thermal environment enhances the entanglement and delays its sudden death phenomenon. The entropic uncertainty $\left(U_{L},U_{R}\right)$ and the two-spin mixedness *L* are enhanced by monotonically increasing the temperature. Furthermore, the function tightness $V_{d}$ starts to emerge from zero and ends when $U_{L}$ and $U_{R}$ overlap, while the relative tightness $U_{d}$ decreases monotonically as the mixedness increases. Figure 1b,c asserts that the evolution of the mixedness is just completely opposite the entanglement. Thus, the mixedness reflects the essence of the entropic uncertainty relations, unlike the strong quantum entanglement destroys the inevitable relationship of the uncertainty.

As a result, we anticipate that, in order to obtain a more accurate measurement between sender Bob and receiver Alice, a quantitative correlation over long distances will be required. It can be determined by the recovery of quantum entanglement between entangled particles under the control of the dipole coupling interactions high value. In addition, the lower bound uncertainty can be used to assess the quality of an uncertain relationship. The smaller the value of the lower bound, the better the quality of uncertainty. Interestingly, the result of a measurement for *R* and *Q* can be better predicted if the lower bound is equal to zero [32].

Figure 1d,f show the dependence of the quantitative memory quantifiers (the entropic uncertainty, the uncertainty tightness, the entropy, and concurrence) on the increase of the thermal environment and the dipole–dipole coupling Δ at $D_{z}=0$. The results of the Figure 1a,d are very similar. We notice that an increase in the critical temperature, when the uncertainty overlaps with all other lower limits, leads to a relative increase in the quantum entanglement, and a relative contrast between the left and right side of the deterministic uncertainty. Thus, the tightness is relatively greater compared to Figure 1a. The mixedness increases from zero to the highest value, then it develops into a constant value with temperature. For the large value of Δ=5, Figure 1 shows that the tightness, the entropic uncertainty relation, and the mixedness can be affected by the increase of the dipole–dipole coupling Δ. The critical temperature at which the quantum entanglement is violated is lower, while the lower limit and the uncertainty have converged, resulting in lower values of the tightness, the relative tightness as well as similar changes in mixedness compared to Figure 1c.

Figure 1f displays the effects of the higher values of the dipole two-spin coupling Δ=ϵ=5 on the entropic uncertainty, the two-spin mixedness and the concurrence under the increase of the temperature. We can deduce that the equal values of the dipole two-spin couplings pairing coefficients suppress the two-spin entanglement completely; see Figure 1b,f. For Δ=ϵ=5, the amplification of the uncertainty and mixedness entropy can be increased clearly, where entropic uncertainty and mixedness grow monotonically through the growth of KT. There are also variations in the relative tightness and the tightness due to the amplification of the gap between the uncertainty due to the increase of the dipole two-spin coupling. This confirms the relation between the correlations and the memory-assisted entropic uncertainty connection. Accordingly, the higher values of the temperature and the dipole–dipole spin system could induce the uncertainty amplification.

**Figure 2:**
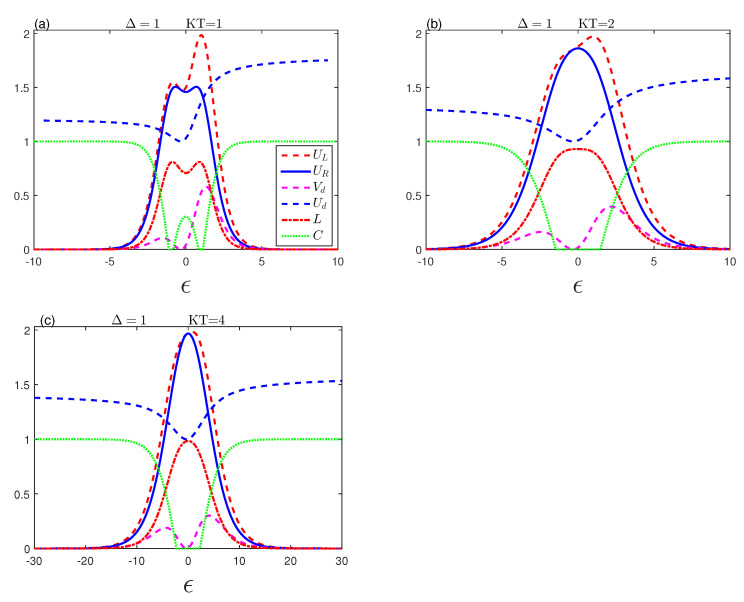
Entropic uncertainty $U_{L}$ (red-dashed line), the lower bound $U_{R}$ (blue-solid line), uncertainty tightness ($V_{d}$ (magenta-dashed line), $U_{d}$ (blue-dashed line)), the mixedness *L* (red dashed-dotted line) and concurrence (green-dotted line) as a function of ϵ with Δ=1 and $D_{z}=0$ for various temperatures.

Figure 2 depicts the effects of the dipolar coupling ϵ on the evolution of the behaviours of the entropic uncertainty. We plot the uncertainty of entropy, the various lower bounds that occurred, the entropic uncertainty relation’s tightness, the mixedness, and the quantum entanglement as a function of the dipolar coupling ϵ for various temperature values in the nonexistence of D–M interaction.

It is clear that the entropic uncertainties, lower bounds, and mixedness increase with |ε|. After that, when the maximum value arrives, they shrink monotonously and reach a minimum at larger enough |ϵ| during fixed temperature states. The entropic uncertainty corresponds to its lower limit at |ϵ|≥4. $U_{R}$ and $U_{L}$ are more synchronized when <0 for KT=1. Meanwhile, we find that the quantum entanglement will vanish for the value ε≈±1 when it is equal to Δ. This means that, when Δ and ϵ are equal, the entanglement is completely destroyed for the system in the absence of D–M, as we explained earlier in Figure 1b. This leads to a discrepancy between $U_{R}$ and $U_{L}$, then entanglement increases to a stable constant value with increasing strength |ε| as in Figure 2a.

In Figure 2b,c, when KT increases, we notice that there is no concurrence, as there are death–birth sudden entanglement intervals around ϵ=0, while the entropic uncertainty relationship approaches its lower bounds and reaches its maximum value and tends to zero with the increase of the coupling ϵ. It is more synchronized with the large coupling force, which differs from its counterpart in Figure 2a. We can see that the strong dipolar interaction decreases the entropic uncertainties, the lower bounds, and mixedness. These quantifiers are highly anti-related to systematic quantum entanglement at low thermal balance temperatures that allow performing accurate measurements.

**Figure 3:**
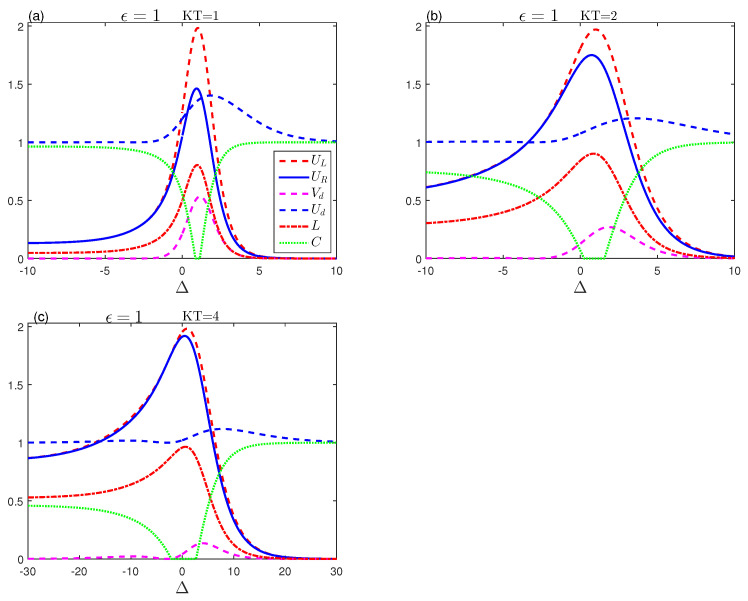
Entropic uncertainty $U_{L}$ (red-dashed line), the lower bound $U_{R}$ (blue-solid line), uncertainty tightness ($V_{d}$ (magenta-dashed line), $U_{d}$ (blue-dashed line)), the mixedness *L* (red dashed-dotted line) and concurrence (green-dotted line) as a function of Δ, for various temperatures KT with ϵ=1 and $D_{z}=0$.

Figure 3 analyzes the general impact of the dipolar coupling Δ on memory-entropic uncertainty, the tightness of the uncertainty, the mixedness, and the entanglement for various thermal resonance temperatures. In this case, the results are qualitatively similar as in Figure 2. For the case where the spins are oriented along the *z*-axis (Δ>0), the amplitudes of the uncertainty and mixedness measures decrease to a minimum value, while entanglement is more stable. In addition, the high temperatures work for the appearance of the death–birth sudden entanglement interval around Δ=0. When the spins are directed at the x−y level (Δ<0), the memory-entropic uncertainty functions have identical behaviour and non-zero values with the weak rotation of the coupling Δ. The amplitudes of these functions can be enhanced by increasing temperature unlike the entanglement. Figure 2 and Figure 3 show that, when the values of Δ of ϵ are small, the tightness $V_{d}$ varies slightly. Higher uniformity of the measuring uncertainty and the bound is reflected by tighter tightness and vice versa. This is evident in the relative tightness $U_{d}$, which indicates, as it approaches 1, that uncertainty and its thresholds are closely related.

**Case $D_{z}\neq 0$**

The following section addresses how $D_{z}$ interaction influences the memory entropic uncertainty, the tightness of uncertainty, the concurrence, and the entropy mixedness in the background of spin systems for dipole–dipole interactions.

**Figure 4:**
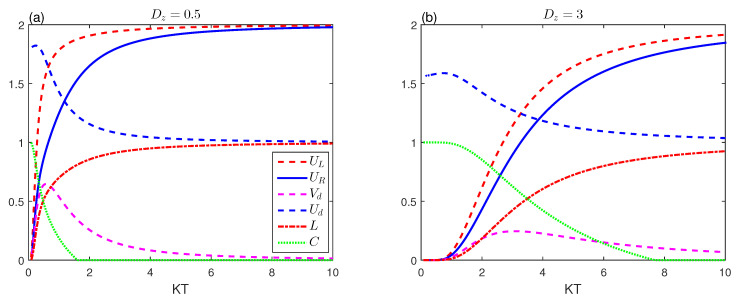
Entropic uncertainty $U_{L}$ (red-dashed line), the lower bound $U_{R}$ (blue-solid line), uncertainty tightness ($V_{d}$ (magenta-dashed line), $U_{d}$ (blue-dashed line)), the mixedness *L* (red dashed-dotted line) and concurrence (green-dotted line) as a function of KT for ϵ=Δ=1 with different values of the parameter $D_{z}$.

Figure 4 depicts the impact of $D_{z}$ interaction on the output of correlations and uncertainty relations, so we plotted the entropic measure of memory uncertainty, uncertainty tightness, mixedness, and concurrence as a function of KT with different values of $D_{z}$ of the D–M interaction: $D_{z}=0.5,3$. It has depicted the developments in various magnitudes, which are similar to those shown in Figure 1b,f. Figure 4 shows that the D–M interaction leads to a revival of the quantum correlation and weakening of the tightness between memory entropic uncertainty relations. Comparing Figure 1b and Figure 4, we observe the monotonously increasing behaviour of both memory entropic uncertainty scales with the temperature. It is clear that the quantitative entanglement can be enhanced due to the increase of the D–M interaction coupling.

**Figure 5:**
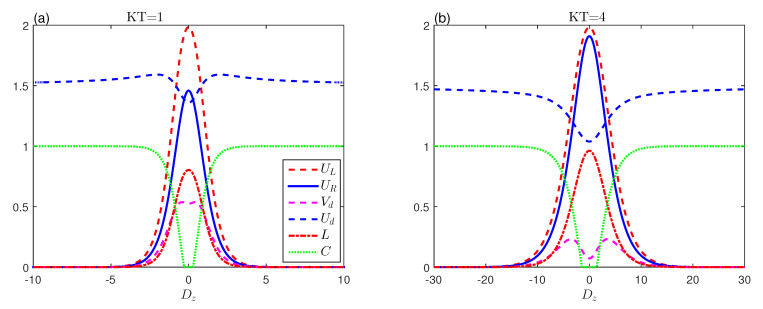
Eentropic uncertainty $U_{L}$ (red-dashed line), the lower bound $U_{R}$ (blue-solid line), uncertainty tightness ($V_{d}$ (magenta-dashed line), $U_{d}$ (blue-dashed line)), the mixedness *L* (red dashed-dotted line) and concurrence (green-dotted line) as a function of $D_{z}$ for various values of KT with ϵ=Δ=1.

Figure 5 shows the effects of the increase of the D–M interaction coupling on the memory entropic uncertainty measures, the tightness of uncertainty, the entropy mixedness, as well as the concurrence entanglement. We find that the entropic uncertainty relations, the lower bound, the tightness of uncertainty, the mixedness, and the concurrence have symmetric behaviour around $D_{z}=0$. The entropic uncertainty relations and the entanglement compatibility are highly sensitive to the effects of the D–M interaction coupling $D_{z}$.

Figure 4 shows that the prosperity of the different quantities seems to be similar to that of Figure 2 with the exception of $D_{z}$ threshold values for different KT constant ratios. This means that the dipole–dipole coupling has the same effects of $D_{z}$ interaction on the quantum memory-entropic uncertainty ($U_{L},U_{R}$), mixedness *L*, tightness $\left(U_{d},V_{d}\right)$ of uncertainty as well as the concurrence. Figure 4a,b reveals that the entropic uncertainty relationships increase monotonically the highest value and then reduce to the lowest value as the strength of $|D_{z}|$ increases. However, the quantum entanglement that is enhanced by increasing the D–M interaction coupling $|D_{z}|$. Furthermore, when $|D_{z}|$ is sufficiently strong, we can see that the uncertainty is significantly reduced. To explain, the entropic uncertainty of quantum memory can be vanishing by reducing the critical values of $|D_{z}|$. This may be significant to ensure that the measurement procedure is conducted correctly. It will enable Bob to identify the effects of simultaneously calculating the observations $\sigma_{x}$ and $\sigma_{z}$. Furthermore, the temperatures reduce, the critical value of $D_{z}$ and the dipole spins’ interactions decrease, and the uncertainties vanish.

**Figure 6:**
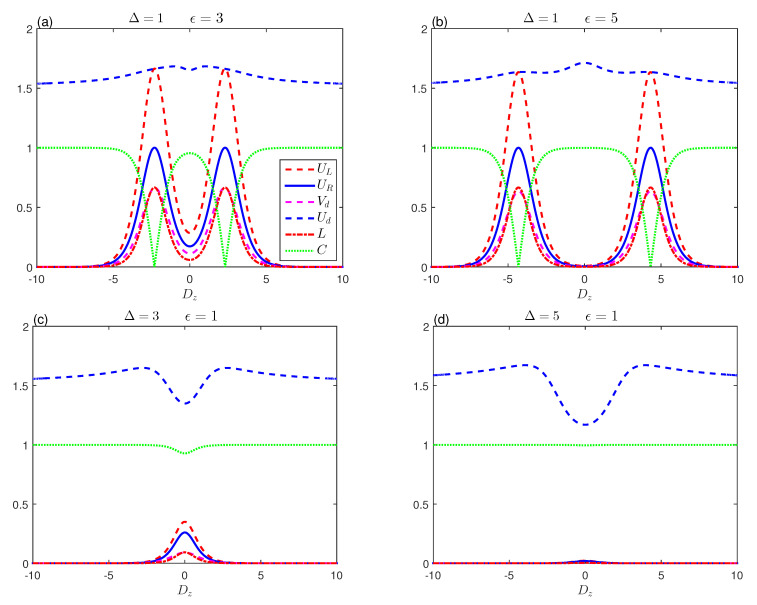
Entropic uncertainty $U_{L}$ (red-dashed line), the lower bound $U_{R}$ (blue-solid line), uncertainty tightness ($V_{d}$ (magenta-dashed line), $U_{d}$ (blue-dashed line)), the mixedness *L* (red dashed-dotted line) and concurrence (green-dotted line) as a function of $D_{z}$ for various values Δ and ϵ with KT=1.

Figure 6a,b clarifies that, when the dipole–dipole parameter ϵ increases, all measurements begin to fluctuate between their maxima and minima until it stabilizes into its stationary values. In Figure 6b, for Δ=1 and ϵ=5, the entanglement and the memory will strongly reduce the measurement results’ uncertainty and for this case the entropic uncertainty is equal to its lower bound at $|D_{z}|$. In Figure 6c,d, the uncertainty and entanglement are plotted as a function of $D_{z}$ for Δ=(3,4) with ϵ=1 and KT=1. We note that the increase of Δ enhances the quantum entanglement and the relative tightness as well as strongly reducing the lower limit of the uncertainty of memory. The uncertainty of memory vanishes when thermal state of the system is reduced.

Figure 6d demonstrates that D–M interaction and the dipole–dipole spin significantly improve prediction of measurement accuracy when Bob and Alice jointly measure the deterministic memory of a two-qubit state when the theoretical deterministic result is zero. For Δ>ε, the maximal entanglement achieves C=1 compared to Figure 6a,b. As a result, the inevitable uncertainty and its lower bound for all moments of the interaction evolves closer to zero, i.e., $U_{L}=U_{R}=0$, allowing Bob to precisely estimate the predictions of Alice’s calculation.

## 5. Conclusions

In this article, we have investigated different features of an entropic uncertainty relation for a dipolar coupled-spin system with D–M interaction in temperature equilibrium, including quantum memory assistance, uncertainty tightening, entanglement, and mixedness. We have analyzed the dipole–dipole spin coupling between two-qubits and the D–M interaction effect on the behavior of entropic uncertainty as temperature rises. The quantum entanglement is diminished at high temperatures, but the uncertainty and mixedness are improved. These investigated quantum measures achieve their stationary values at increasing temperature levels. According to the results, mixedness is closely connected to the entropic uncertainty, and they behave similarly with different amplitudes. It is found that, in the absence of D–M interaction, the sudden death entanglement phenomenon is caused by equal dipolar two-spin interactions. Mixedness has a significant impact on the relationships of uncertainty and tightness, as well as the accuracy of the measurement between Alice and Bob. We discovered that the Dzyaloshinsky–Moriya interaction at a specific temperature, as well as the dipole interaction coupling, can suppress uncertainty relations and characterize the emergence of entanglement to the maximum extent possible. In the absence of D–M interaction, it was observed that the sudden death entanglement phenomenon appears due to being equal to the dipolar two-spin interactions. Bob can then properly predict and acquire Alice’s result in this situation. Our research could open up a new window into the dynamical evolution of entropic uncertainty relations in quantum spin models of asymmetric dipole–dipole spin interaction, where a dipolar coupled-spin system of Alice and Bob is possible, for predicting measurement accuracy in quantum information processing. 

## Data Availability

Not applicable.
